# Social inclusion inequalities between individuals with mental disorders and the general population: a systematic review and meta-analysis

**DOI:** 10.1017/S2045796026100730

**Published:** 2026-06-10

**Authors:** Giulia Pollice, Mattia Marchi, Maria Trapani, Alice Pagnucco, Silvia Ferrari, Luca Ghirotto, Luca Pingani, Gian Maria Galeazzi

**Affiliations:** 1 Department of Biomedical, Metabolic and Neural Science, University of Modena and Reggio Emilia, Modena, Italy; 2 Dipartimento ad Attività Integrata Salute Mentale e Dipendenze Patologiche, Azienda USL-IRCCS di Reggio Emilia, Reggio Emilia, Italy; 3 Department of Philosophy, Sociology, Education and Applied Psychology, University of Padua, Padua, Italy; 4 Qualitative Research Unit, Azienda USL-IRCCS di Reggio Emilia, Reggio Emilia, Italy

**Keywords:** community mental health, epidemiology, social factors, social inclusion, systematic reviews

## Abstract

**Aims:**

Social inclusion is increasingly recognised as a key determinant of health and well-being, encompassing participation in social, economic, political and cultural life through access to resources, opportunities and relationships. Individuals with mental disorders are at increased risk of social exclusion, yet existing evidence often relies on broad population-level indicators that fail to capture the multidimensional nature of inclusion. This systematic review aimed to compare levels of social inclusion between these groups using validated psychometric instruments and to identify the dimensions in which disparities are most pronounced.

**Methods:**

The review followed PRISMA guidelines. A two-step search strategy was conducted across PubMed, Embase, PsycINFO, Scopus, CINAHL and Web of Science. First, validated measures of social inclusion and related constructs were identified. Second, studies applying these instruments in individuals with and without mental disorders were retrieved. Eligible studies included adults with clinician-established or self-reported diagnosis of mental disorders and comparison groups from the general population. A random-effects meta-analysis was conducted to estimate standardised mean differences (SMDs) with 95% confidence intervals (CIs) between the two groups, while a thematic narrative synthesis explored domain-specific inequalities.

**Results:**

Ten studies met inclusion criteria, and six were included in the meta-analysis, comprising 844 individuals with mental disorders and 1086 controls. Individuals with mental disorders reported significantly lower levels of social inclusion than the general population (SMD = −0.91; 95% CI: −1.25 to −0.56). The narrative synthesis identified inequalities across several interconnected domains. Individuals with mental disorders experienced weaker social relationships, lower perceived support, reduced community participation and fewer opportunities for meaningful engagement. Marked disadvantages were also observed in employment, income, education and housing, including financial hardship, insecure living conditions and neighbourhood dissatisfaction. Several studies highlighted discrepancies between objective indicators of participation and subjective experiences of inclusion, indicating that participation alone may not reflect a sense of belonging or access to valued social roles. Socioeconomic position, gender and ethnicity appeared to intensify exclusion across domains.

**Conclusions:**

Individuals with mental disorders experience substantial inequalities in social inclusion across interconnected relational, community and socioeconomic domains. These disparities are shaped by broader structural conditions and compounded by intersecting forms of disadvantage, including socioeconomic position, gender and ethnicity. The findings highlight the need for multidimensional, intersectional and person-centred approaches that recognise both the cumulative nature of exclusion and individuals’ subjective experiences of inclusion. Policies and interventions should address the social determinants that constrain opportunities for meaningful inclusion.

OSF: https://doi.org/10.17605/OSF.IO/USXWG.

## Introduction

Social inclusion is increasingly recognised as a fundamental determinant of health and well-being. It refers to the extent to which individuals can participate in economic, social, political and cultural life through access to relevant resources, opportunities and relationships (Popay, 2010). Research indicates that individuals living with mental disorders face markedly higher risks of social exclusion than the general population (Boardman, 2011). This risk is consistently associated with poorer health outcomes, restricted opportunities and reduced quality of life (Killaspy *et al.*, 2014). Yet, much of the existing evidence relies on broad survey indicators or population-level correlational data, which often fail to capture the multidimensional nature of inclusion and exclusion, limiting the understanding of how these processes operate and which specific domains are most affected.

Conceptually, social inclusion has been described as both an outcome and a dynamic process enabling participation, while social exclusion has been defined as an ‘enforced lack of participation’, involving material deprivation, reduced agency and experiences of marginalisation and inequality (Morgan *et al.*, 2008). *Relational* approaches further emphasise that exclusion arises from unequal power relations, producing a continuum rather than a binary distinction between inclusion and exclusion (Popay, 2010). Despite its widespread use in policy and practice, social inclusion remains difficult to operationalise, as its indicators frequently function simultaneously as determinants and outcomes of inclusion (Ponce and Rowe, 2018).

These conceptual challenges are particularly salient in mental health. Individuals living with severe mental disorders are among the groups most exposed to social exclusion (Marmot, 2018), experiencing reduced access to employment, income, housing stability, social networks and community participation, alongside heightened exposure to discrimination and stigma (Boardman, 2011). Such patterns are not uniform and may be more pronounced among individuals experiencing multiple forms of disadvantage, including those related to ethnicity or socioeconomic position (Kirkbride *et al.*, 2024). Evidence suggests a bidirectional relationship whereby disadvantage may contribute to the onset of mental disorders, while illness trajectories can further constrain opportunities for participation, especially within disabling social arrangements, resulting in cumulative disadvantage over time (Eager *et al.*, 2025). These processes are also reflected in patterns of service access and intervention, with socioeconomic position shaping pathways to care and potentially contributing to unequal outcomes (Barnett *et al.*, 2023).

Against this background, social inclusion has become a key framework for understanding lived experience, recovery trajectories and public responsibilities in mental health. Recent reviews have synthesised evidence on inclusion-oriented practices in severe mental disorders, highlighting both their growing prominence and persistent gaps between policy aspirations and service delivery (Henderson *et al.*, 2026). In parallel, the need for more precise and multidimensional assessment has led to the development of psychometric instruments designed to capture social inclusion across multiple life domains, with greater attention to individuals’ subjective experiences (Cordier *et al.*, 2017).

Despite the availability of validated measures, no review has systematically compared social inclusion between individuals with mental disorders and the general population. Existing studies have focused on single samples, specific populations or scale validation, leaving two major gaps: a pooled estimate of the magnitude of inequality and a domain-specific understanding of where disparities are most pronounced.

This review addresses these gaps by:

- Quantifying differences in social inclusion between individuals with and without mental disorders using validated psychometric instruments.
- Synthesising domain-specific findings to identify patterns and interpret them within relevant theoretical and sociocultural frameworks.

Together, these approaches clarify the extent and nature of inequalities and their implications for mental health research and practice.

## Methods

The review followed the PRISMA guidelines (Page *et al.*, 2021). The study protocol was registered on the Open Science Framework (https://doi.org/10.17605/OSF.IO/USXWG).

### Search strategy

We first identified psychometric instruments used to quantitatively assess social inclusion, then conducted a second search to identify studies applying these instruments to compare individuals with mental disorders and the general population.

Searches were structured around the construct of interest, the target population and measurement-related filters and were run in PubMed, CINAHL, Scopus, Embase and PsycINFO, with no language or time restrictions applied. Search strings adapted for each database are reported in Supplementary Materials, Section 1. Each identified scale was then searched individually within the same databases to retrieve studies in which it had been applied both in individuals with mental disorders and in the general population; this process was completed in November 2025. Three reviewers independently screened records; disagreements were resolved by discussion, consulting a fourth reviewer when needed.

### Inclusion criteria

We included scales assessing social inclusion or closely related constructs (e.g., social or community integration, community participation) that had been used and validated in the target population, where the basis of diagnosis was either clinician-established or self-reported by participants as a previously received diagnosis. Studies focusing on children or older adults were excluded. All questionnaire formats were eligible, whether self-administered or completed with assistance, and only full-text, peer-reviewed articles were included. The selection of social inclusion measures is described in detail in Supplementary Materials, Section 2.

Multiple publications based on the same sample were retained for the narrative synthesis, while for the meta-analysis, only one publication per sample was included, prioritising the report with the most complete data (see Supplementary Materials, Section 3).

### Data extraction

For each eligible comparison, two reviewers independently extracted data on study and sample characteristics, social inclusion measures, study aims and main findings, group means and standard deviations, and – where available – domain-specific group differences and authors’ interpretations. Extraction sheets were cross-checked, and disagreements were resolved through discussion.

Risk of bias was assessed using the Cochrane risk of bias tool (Higgins *et al.*, 2011). The certainty of the evidence for the pooled estimate was assessed using the GRADE framework (Guyatt *et al.*, 2008).

### Data analysis and synthesis

#### Meta-analysis

Where studies reported disaggregated data (e.g., total-scale means and standard deviations), we performed meta-analysis using random-effects models.

We calculated standardised mean differences (SMDs; Hedges’ *g*) as the difference in social inclusion score between individuals with mental disorders and the general population, with 95% confidence intervals (95% CIs) and quantified heterogeneity using Cochran’s *Q, τ*^2^ and *I*^2^ (Higgins and Thompson, 2002). The results were summarised using forest plots. Robustness was assessed using leave-one-out analyses.

To explore heterogeneity, we examined the contribution of clinical severity using (i) a random-effects subgroup analysis comparing studies including only individuals with severe mental disorders with studies including mixed-severity samples, and (ii) a meta-regression with the proportion of participants with psychosis as a continuous moderator. Analyses were conducted in R (version 4.4.2) using the metafor and meta packages (Viechtbauer, 2025).

#### Narrative synthesis

A narrative synthesis was conducted using thematic analysis to examine how differences between the two groups were described and operationalised across studies and to identify the domains in which disparities were most pronounced. The analysis followed a deductive–inductive approach (Thomas and Harden, 2008). Initial coding was deductive and based on dimensions reported in the included studies, with inductive refinement used to reorganise and expand these dimensions where emerging patterns required further specification. Two reviewers independently developed an interpretative coding framework to classify recurrent domains of social inclusion.

## Results

### Overview of the included studies

**Figure 1.**
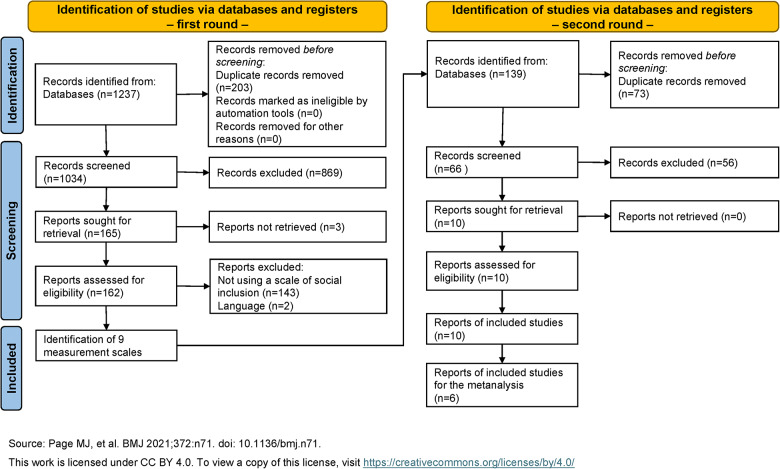
PRISMA flow chart. Figure 1 long description.

Following the PRISMA flow diagram, 10 reports were identified (Figure 1), 6 of which provided data suitable for inclusion in the meta-analysis. Ten studies employed validated social inclusion measures to compare individuals with and without mental disorders.

Table 1 summarises studies’ aims and key findings. Across all studies, individuals with mental disorders consistently reported substantially lower levels of social inclusion. However, studies differed in the domains emphasised: some focused mainly on community participation, whereas others adopted broader multidimensional approaches encompassing social networks, economic inclusion, social participation and community support. A detailed overview of the scales used and their corresponding domains of assessment is provided in Supplementary Materials, Section 4. Most studies were conducted in Western countries, while non-Western contexts were represented primarily through cross-cultural comparative studies.

**Table 1.** Characteristics of the studies: country, scale, aims and key findings Table 1 long description.

Study	Country	Scale	Aims	Key findings
Aubry and Myner, 1996	Canada	Community Inclusion Scale (CIS)	Comparing levels of community integration between a group of individuals with mental disorders living in supported housing programmes with the general population from the surrounding neighbourhood.	Individuals with mental disorders reported lower social integration and quality of life than community residents, particularly in social relationships.
Cabral *et al*., 2018	Portugal	Community Inclusion Scale for Adults with Psychiatric Problems (CIS-APP-34)	Validating the scale	Lower social inclusion among individuals with mental disorders across all domains, except community support.
Filia *et al*., 2019	Australia	Filia Social Inclusion Measure (F-SIM)	Validating the scale and comparing three samples: people with mental disorders, caregivers and individuals from the general population.	Individuals with mental disorders reported lower scores across all domains of social inclusion relative to the general population group, whereas caregivers showed intermediate levels.
Filia *et al*., 2022	Australia	Filia Social Inclusion Measure (F-SIM)	Validating the scale and comparing five groups: young people with psychosis, young people with other severe mental disorders, adults with severe mental disorders and two age-matched control groups	Individuals with mental disorders showed consistently lower social inclusion than the general population; young people with psychosis were particularly disadvantaged.
Gardner *et al*., 2019	Australia	Filia Social Inclusion Measure (F-SIM)	Comparing social inclusion among young adults (18–25 years) with severe mental disorders and peers across three domains	Young adults with severe mental disorders reported lower participation in work and education, weaker relationships and greater housing instability.
Huxley *et al*., 2012	UK	Social and Community Opportunities Profile (SCOPE)	Validating the scale	The SCOPE subscale ‘Satisfaction with opportunities’ was significantly lower in the service users’ sample.
Huxley *et al*., 2016	UK, HK	Social and Community Opportunities Profile (SCOPE)	Cross-cultural validation of the scale	UK patient sample reported the lowest level of social inclusion, the Hong Kong patient sample showed an intermediate level and the UK general population sample displayed the highest perceived inclusion.
Mezey *et al*., 2020	UK	Social Inclusion Questionnaire – User Experience (SINQUE)	Validating the scale	Marked reduction in social inclusion among individuals with mental disorders compared with siblings, especially in social integration and productivity.
Nagata *et al*., 2020	USA	Temple University Community Participation Measure (TUCP)	Comparing social inclusion between individuals with severe mental disorders and the general population	Lower community participation among individuals with severe mental disorders
Santos *et al*., 2018	UK, HK, Brazil	Social and Community Opportunities Profile (SCOPE)	Comparing international results	Lower social inclusion in clinical samples, strongly shaped by structural inequalities (education, income, gender, ethnicity).

UK, United Kingdom; HK, Hong Kong; USA, United States of America.

Table 2 summarises sample characteristics. Sample sizes, clinical profiles and demographic composition varied across studies. Most participants with mental disorders had severe or long-term conditions and were recruited from diverse settings, including community mental health services, supported housing programmes and broader community contexts. Women were generally over-represented in both groups. Comparison samples included community members, caregivers or family members.

**Table 2.** Characteristics of the samples Table 2 long description.

		Sample of people with mental disorders			Sample of people from general population		
Reference	Country	*n*	Clinical info	Demographic	*n*	General info	Demographic
Aubry and Myner, 1996	Canada	51	**Diagnosis: severe MD** (Clinician-diagnosed [service-based]) 57% schizophrenia, 14% affective disorders; **Admission *n*°**: 4 (mean); **Length of hospital stay** (mean months): 30; Other info: all in a housing programme	NA	51	NA	NA
Cabral *et al.*, 2018	Portugal	183	**Diagnosis**: (Clinician-diagnosed [service-based]) NA	**Age**: 44.26 (sd 13.51) **Gender**: 72.7% F **Ethnicity**: NA	228	NA	**Age**: 42 (sd 13.95) **Gender**: 71.1% F **Ethnicity:** NA
Filia *et al.*, 2019	Australia	42	**Diagnosis:** (Clinician-diagnosed [service-based]) Depression ∼ 30%, Bipolar ∼ 17%, Schizophrenia ∼ 30%, Personality ∼ 13%	**Age**: 40.37 (sd 9.87) **Gender**: 73.3% F **Ethnicity**: NA	86	49: carers, 37: community members	**Age**: 45.27 (sd 14) carers 36.6 (sd 10) community **Gender**: 80% F carers; 60% F community members **Ethnicity:** NA
Filia *et al.*, 2022	Australia	239	**Diagnosis: severe MD** (Clinician-defined [not specified]) 149 = young with psychosis; 26 = young with other MD; 64 = older with MD	**Age**: 175 aged <26, 64 aged >26 **Gender: ∼**45% F (aged <26), ∼58% (aged >26) **Ethnicity**: NA	267	NA	**Age**: 163: <26 years; 104: >26 years **Gender: ∼**53% F (<26), ∼65% F (>26) **Ethnicity:** NA
Gardner *et al.*, 2019	Australia	159	**Diagnosis: severe MD** (Self-report [of clinician diagnosis]) 53% depression, 43% anxiety, 30% schizophrenia	**Age**: 18–25 **Gender**: 48.4% F **Ethnicity**: NA	152	Wide range of community settings	**Age:** 18–25 **Gender**: 51.3% F **Ethnicity:** NA
Huxley *et al.*, 2012	UK	43	**Diagnosis: severe MD** (Clinician-diagnosed [service-based])	**Age**: 49 (sd 12) **Gender**: 56% F **Ethnicity**: NA	212	Random from postal address files	**Age**: 55 (sd 21) **Gender**: 57.5% F **Ethnicity:** NA
Huxley *et al.*, 2016	UK, HK	211 (43 UK, 168 HK)	**Diagnosis:** (Clinician-diagnosed [service-based]) Long-term disorder 92% UK, 49% HK	**Age**: UK 48% aged <50, HK 59% aged <50 **Gender**: UK 56% F; HK 52% F **Ethnicity**: NA	212 UK	NA	**Age**: NA **Gender**: 41% F **Ethnicity:** NA
Mezey *et al.*, 2020	UK	192 (28 compared with their siblings)	**Diagnosis**: (Clinician-diagnosed [clinical records]) 55% psychosis, 26% common MD, 19% personality **Duration of contact**: 17.5 years (sd 10.6) **Involuntary admissions**: 4.8 (sd 6.9)	**Age**: 42.2 (sd 11.4) **Gender**: 56% F **Ethnicity**: 67% White	28	Sibling of people with MD	**Age**: 40.3 (sd 12.9) **Gender**: 57% F **Ethnicity**: 79% White
Nagata *et al.*, 2020	USA	300	**Diagnosis: severe MD** (Self-report [of clinician diagnosis]) Mood disorder 57%; Schizophrenia 43%	**Age**: 46.3 (sd 11.23) **Gender**: 60% F **Ethnicity**: 60% white, 25% black, 1% Asian, 4% Hispanic, 1% Native American and 9% identified as other	300	NA	**Age**: 51.5 mean (sd 11.33) **Gender**: 55% F **Ethnicity**: 79% white, 10% black, 1% Asian, 3% Hispanic, 1% Native American and 6% identified as other
Santos *et al.*, 2018	Brazil, UK, HK	225 Brazil	45% chronic illness (Clinician-diagnosed [implied, not specified])	**Age**: 56% <40 **Gender**: 52% F **Ethnicity**: White 53%, Black 12.4%, Mixed 27.6%, Asian 2.7%, Indigenous 4.4%	212 UK	NA	**Age**: 32% under 40 **Gender**: 57.5% F **Ethnicity**: 100% white
		168 HK	49.4% chronic illness	**Age**: 40% <40 **Gender**: 47.6% F **Ethnicity**: Asiatic 100%			

MD, mental disorder; F, female; sd, standard deviation; UK, United Kingdom; HK, Hong Kong; USA, United States of America.

The certainty of the evidence was rated as low, starting from observational studies, downgraded for risk of bias and substantial heterogeneity, and partially offset by an upgrade reflecting the magnitude and consistency of the pooled effect. Further details are provided in Supplementary Materials, Section 5.

### Meta-analysis

**Figure 2.**
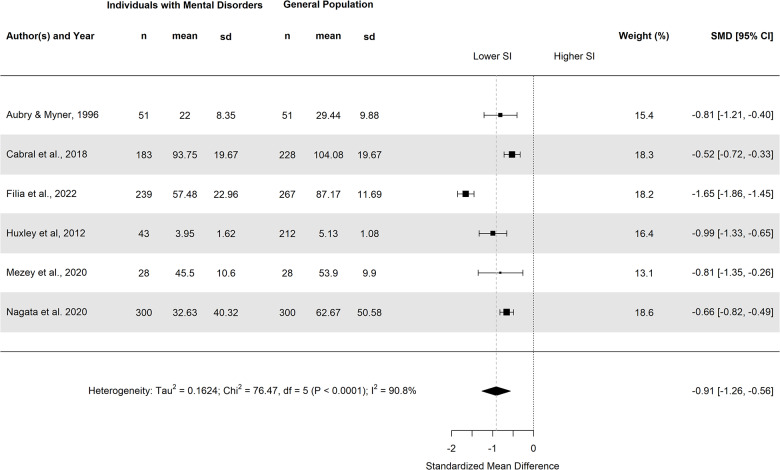
Forest plot. Figure 2 long description.

Six studies provided data suitable for meta-analysis, comprising 844 individuals with mental disorders and 1086 from the general population. The pooled effect showed substantially lower levels of social inclusion among individuals with mental disorders (SMD = −0.91, 95% CI: −1.25; −0.56). Between-study heterogeneity was high (*I*^2^ = 90.8%) and statistically significant (*p* < .0001). However, all study-level estimates were directionally consistent, as shown in Figure 2.

*n*, number; sd, standard deviation; SI, social inclusion. Higher scores indicate higher levels of social inclusion

Leave-one-out analyses confirmed the robustness of the pooled estimate, as all iterations showed effects in the same direction and of similar magnitude. Detailed results are reported in Supplementary Materials, Section 6.

The random-effects subgroup analysis showed a more negative pooled effect in studies including only individuals with severe mental disorders (SMD = −1.03, 95% CI: −1.49 to −0.58) compared with studies including mixed clinical severity (SMD = −0.56, 95% CI: −0.74 to −0.37). However, the CIs for the between-group comparison were wide and included the null, indicating uncertainty in the magnitude of this difference.

Meta-regression using the proportion of participants with psychosis as a continuous moderator yielded a negative coefficient (*β* = −0.044, 95% CI: −0.106 to 0.017), although the CI included zero. The model explained 36.11% of between-study heterogeneity (*R*^2^ = 36.11%), with significant residual heterogeneity remaining (*p* = .008).

Overall, these analyses suggest a tendency towards greater disadvantage in social inclusion with increasing clinical severity but do not provide precise estimates of its contribution to variability in effect sizes.

### Narrative synthesis results

The thematic analysis identified five overarching domains, summarised in Table 3.

**Table 3.** Thematic synthesis of social inclusion domains Table 3 long description.

Domain	Findings	Reference
Social connections	Individuals with mental disorders showed weaker and less supportive networks than the general population.	Filia *et al.*, 2019, 2022; Gardner *et al.*, 2019; Huxley *et al.*, 2012; Mezey *et al.*, 2020; Santos *et al.*, 2018; Aubry and Myner, 1996
	Compared to the general population, for individuals with mental disorders, contact with neighbours was rare or occasional.	Aubry and Myner, 1996
	Increased reliance on digital interaction was observed among individuals with mental disorders (60.0% vs 23.3%; *χ*^2^ = 9.07, *p* = .011).	Filia *et al.*, 2019
	Young people with severe mental disorders were five times less likely to perceive having friends available to support them (OR = 0.19, 95% CI: 0.04, 0.52) and felt less able to provide support to their friends (OR = 0.19, 95% CI: 0.04–0.53)	Gardner *et al.*, 2019
	Longitudinal data showed a widening gap in social integration over time between individuals with mental disorders and their unaffected siblings, and the difficulties in social inclusion may precede clinical onset.	Mezey *et al.*, 2020
	Family relationships were strongly shaped by socioeconomic disadvantage, with lower income and education associated with reduced familial acceptance and support for the person with a mental disorder (*F* = 4.41, *p* < .001)	Santos *et al.*, 2018
Community integration and social participation	Community participation showed the largest gap, with individuals with mental disorders consistently reporting lower levels of engagement.	Cabral *et al.*, 2018; Mezey *et al.*, 2020; Huxley *et al.*, 2016
	The breadth and diversity of participation were reduced, reflecting narrower patterns of engagement across everyday and social activities (mean 6.95 vs 8.67; *F* = 11.86, *p* < .001).	Nagata *et al.*, 2020
	Socioeconomic adjustments reduced the *objective* disparity, indicating that material conditions account for part – but not all – of the difference (50.58 vs 62.67; *t* = −4.03, *p* < .001; adjusted means 56.97 vs 55.01; income: *F* = 15.62, *p* < .001; car ownership: *F* = 8.81, *p* = .003).	Nagata *et al.*, 2020
	Structural inequalities related to race, gender, education and income compounded exclusion, producing stratified patterns of social participation among people with mental disorders (e.g., *leisure domain: χ*^2^ = 17.56 by gender; *F* = 8.10 by education; *F* = 3.46 by income; *F* = 2.46 by ethnicity; *p* < .05).	Santos *et al.*, 2018
	Subjective perceptions and satisfaction with participation and opportunities for participation were substantially poorer among individuals with mental disorders, even when objective indicators appear comparable.	Nagata *et al.*, 2020; Filia *et al.*, 2019; Huxley *et al.*, 2012, Huxley *et al.*, 2016; Aubry and Myner, 1996
	Family members exhibited intermediate outcomes, performing better than individuals with mental disorders but not reaching the levels reported by the general population.	Filia *et al.*, 2019
Income, employment and education accessibility	Significant disparities in income and financial security were reported across studies, with individuals with mental disorders experiencing lower earnings, greater financial strain and prolonged poverty.	Filia *et al.*, 2019, 2022; Gardner *et al.*, 2019
	Financial hardship restricted participation in leisure (*χ*^2^ = 6.48), holidays (*χ*^2^ = 13.44) and social activities (*χ*^2^ = 7.27), thereby further limiting community inclusion.	Filia *et al.*, 2019
	Individuals with mental disorders faced poorer employment opportunities (Filia; *χ*^2^ = 7.47), often confined to sheltered work, indicating partial and welfare-mediated economic inclusion.	Filia *et al.*, 2019; Santos *et al.*, 2018
	Longitudinal evidence shows worsening outcomes over time, with individuals with mental disorders displaying lower productivity at follow-up compared with their siblings, despite no initial pre-onset differences.	Mezey *et al.*, 2020
	Disparities in work-related inclusion were strongly influenced by education and income, with marked gender inequalities (*work domain*: gender: *χ*^2^ = 33.04; education: *F* = 4.30; income: *F* = 7.52; *p* < .05)	Santos *et al.*, 2018
	Lower levels of formal educational attainment among individuals with mental disorders. For instance, in Gardner *et al.* (2019), young adults with severe mental disorders were more than two times less likely to be currently studying at a formal institution (OR = 0.44, 95% CI: 0.30, 0.64).	Cabral *et al.*, 2018; Gardner *et al.*, 2019; Mezey *et al.*, 2020; Santos *et al.*, 2018
Housing and living conditions	Individuals with mental disorders were more likely to live alone, express dissatisfaction with their neighbourhood and report greater exposure to crime and violence.	Filia *et al.*, 2019; Santos *et al.*, 2018
	Individuals with mental disorders reported lower housing costs (*F* = 5.61), alongside higher rates of living alone (*χ*^2^ = 9.12) and neighbourhood crime exposure (*χ*^2^ = 8.96).	Filia *et al.*, 2019
	Individuals with severe mental disorders and low income were more likely to live with their families (Gardner; OR = 0.19, 95% CI: 0.04, 0.52).	Santos *et al.*, 2018; Gardner *et al.*, 2019
	Non-white individuals and/or with a lower level of education and/or income reported poorer housing conditions and higher neighbourhood insecurity, illustrating entrenched socio-spatial inequalities (*housing domain*: ethnicity *F* = 2.57; *neighbourhood safety domain*: education *F* = 3.48, income *F* = 3.02; *p* < .05).	Santos *et al.*, 2018
	Compared to peers, young people with severe mental disorders were five times more likely to live with their parents and three times more likely to experience housing instability (OR = 3.58, 95% CI: 1.14, 11.15).	Gardner *et al.*, 2019
	Congregate living settings in disadvantaged neighbourhoods, while providing shelter, did not promote meaningful neighbour relationships and may have reinforced symbolic segregation, as community residents showed higher levels of social integration even after adjustment for physical integration (*F*(1,50) = 18.96, *p* < .001).	Aubry and Myner, 1996
Political and civic inclusion	No significant differences between individuals with mental disorders and their non-affected siblings in terms of access to public services (mean difference: −0.2, 95% CI: −0.7 to 0.4) or political participation (mean difference: 0.0, 95% CI: 0.0–0.0).	Mezey *et al.*, 2020

**1. Social connections**

Across studies, social networks – including family, friends and informal ties – were weaker among individuals with mental disorders. Studies reported pronounced relational vulnerability, including lower perceived availability of friends for support in times of crisis (Gardner *et al.*, 2019; Filia *et al.*, 2022). Differences concerned not only the number of social contacts but also the perceived reciprocity and supportive quality of relationships, including the ability to support others (Gardner *et al.*, 2019). Longitudinal evidence suggested that lower levels of social integration were already present prior to first service contact (Mezey *et al.*, 2020). At the neighbourhood level, individuals with mental disorders reported fewer and more sporadic contacts with neighbours, highlighting a gap between physical proximity and meaningful social embeddedness (Aubry and Myner, 1996). Finally, family relationships appeared shaped by structural disadvantage: lower income and educational attainment were associated with reduced familial acceptance and support, potentially intensifying isolation and dependency (Santos *et al.*, 2018).

**2. Community integration and social participation**

Individuals with mental disorders consistently reported lower community participation, with this domain often showing one of the largest between-group differences. Evidence suggested that part of this gap was related to socioeconomic conditions. After adjustment for material resources, such as income and car ownership, objective participation no longer differed between groups (Nagata *et al.*, 2020). Participation patterns were instead shaped by intersecting indicators of social position, including education, income, ethnicity and gender, influencing political, social and recreational engagement as well as satisfaction with leisure activities (Santos *et al.*, 2018).

However, differences persisted in subjective experiences of participation. Individuals with mental disorders reported lower satisfaction with community engagement and involvement in a narrower range of activities, even after adjustment for socioeconomic factors (Nagata *et al.*, 2020), with perceived lack of opportunities emerging as a strong indicator.

**3. Income, employment and education opportunities**

*Income*

Across all included studies, individuals with mental disorders reported lower income levels than the general population. Studies also highlighted pronounced financial hardship, including persistent economic strain, prolonged poverty and difficulties meeting essential expenses. These constraints limited participation in leisure, holidays and social activities, thereby reinforcing exclusion (Filia *et al.*, 2019, 2022).

*Employment*

Employment rates among individuals with mental disorders were consistently lower than those in general population samples, often by a substantial margin (9–20% vs 75–91%) (Mezey *et al.*, 2020). Even individuals with recent work, education or training experience reported greater occupational limitations, largely attributed to restricted opportunities and insufficient skills or qualifications (Filia *et al.*, 2019). Findings from Mezey et al. suggested that these inequalities may intensify over time: while no differences in productivity were observed prior to illness onset, individuals with mental disorders showed lower productivity than non-affected siblings after prolonged service contact. An exception was observed in the Hong Kong sample, where employment levels were relatively high, however, largely confined to sheltered or protected settings, indicating welfare-mediated labour market inclusion (Santos *et al.*, 2018).

*Education*

Lower formal educational attainment among individuals with mental disorders was reported across multiple national contexts, with clinical samples more likely to report very low schooling or no qualifications beyond basic education (Cabral *et al.*, 2018; Santos *et al.*, 2018). In the UK, a substantially smaller proportion had completed A-level qualifications compared with their non-affected siblings (25% vs 75%) (Mezey *et al.*, 2020).

**4. Housing and living conditions**

Housing conditions among individuals with mental disorders were often precarious, with a higher likelihood of living alone, neighbourhood dissatisfaction and exposure to crime and insecurity. These vulnerabilities were patterned by socioeconomic and demographic factors, with lower income, non-white ethnicity and lower educational attainment associated with greater perceived insecurity and housing-related exclusion (Santos *et al.*, 2018). Lower housing costs often reflected poorer quality rather than affordability (Filia *et al.*, 2019). Earlier evidence also showed that many individuals with mental disorders lived in congregate housing in disadvantaged neighbourhoods characterised by high turnover and low social cohesion. Although such arrangements provided basic shelter, they did not consistently support meaningful neighbour relationships and may contribute to symbolic forms of segregation (Aubry and Myner, 1996).

**5. Political and civic inclusion**

Evidence on civic and political participation was limited. The only study addressing this domain found no significant differences in access to services or political engagement, suggesting these areas may be less sensitive to mental health status or more uniformly accessible across groups (Mezey *et al.*, 2020).

## Discussion

The meta-analysis indicated a clear disadvantage for individuals with mental disorders, who reported lower levels of social inclusion than the general population. Despite substantial heterogeneity, effects were consistent in direction and of medium-to-large magnitude. Notably, despite the overall low certainty rating, the consistency and magnitude of the observed effect strengthen confidence in the direction of the association. These findings align with literature describing a close, potentially bidirectional association between social exclusion and mental disorders (Boardman, 2011). Exploratory analyses suggested a tendency towards greater disadvantage in social inclusion with increasing clinical severity, consistent with previous literature. However, estimates were imprecise, and CIs included the null value, indicating uncertainty in the magnitude of this difference. In line with existing literature, this pattern may reflect the complex interplay between clinical severity and broader social and structural factors, including greater exposure of more severe conditions to structural stigma (Evans-Lacko *et al.*, 2014; Killaspy *et al.*, 2014; Eager *et al.*, 2025). The thematic synthesis further showed that inequalities extend across interpersonal and relational domains, socioeconomic position, community participation and living conditions. Structural factors related to education, ethnicity and gender appeared to intensify these patterns, while evidence on civic and political inclusion was limited. Overall, the findings indicate that lower levels of social inclusion are cumulative, structurally patterned and expressed across interconnected domains of everyday life.

### Social and community dimensions of social inclusion

Social connections emerged as a particularly affected domain. Across studies, individuals with mental disorders reported poorer relationship satisfaction, fewer perceived opportunities for connection and lower reciprocity within social networks. These findings align with the conceptualisation of social connection as a construct comprising structural aspects of networks, the functional role of support and the quality of relationships, each differentially influencing mental health outcomes (Hajek *et al.*, 2025). Consistent with this perspective, longitudinal studies indicate that limited perceived support and persistent difficulties in maintaining social ties are associated with less favourable recovery trajectories and poorer quality of life (Hajek *et al.*, 2025).

However, while social relationships constitute a core component of social inclusion, they do not fully capture its broader scope (Simplican *et al.*, 2015). Community participation represents a more complex layer, involving access to valued social roles, opportunities for engagement and legitimacy within community settings. Across studies, individuals with mental disorders frequently reported low satisfaction with participation in community life and perceived limited opportunities to engage as active members, even when some objective opportunities were available. Such dynamics resonate with the notion of ‘program citizenship’, whereby participation remains conditional, service-bound and only partially extends to the wider community (Ponce and Rowe, 2018).

Sociodemographic factors, including gender, ethnicity and income, further shaped these experiences, producing stratified forms of community inclusion. These patterns reflect structural marginalisation associated with these *social locations*, resulting in unequal distribution of power and opportunities (Collins, 2002). Such inequalities are manifested in conditions such as living in unsafe neighbourhoods, financial constraints and community stigma, which in turn constrain opportunities for meaningful community participation (Salzer, 2021).

### Socioeconomic conditions and structural inequalities

A further salient finding concerns disparities in social, economic and cultural rights, including access to adequate housing, education and dignified employment. Across studies, individuals with mental disorders experienced persistent economic disadvantage, restricted educational and employment opportunities, limited access to basic goods and precarious housing conditions, compounding and sustaining processes of exclusion (Marmot, 2018). Consistent with existing evidence, individuals with mental disorders often reside in housing they consider unsatisfactory, within marginalised neighbourhoods where they do not feel safe (Fossey *et al.*, 2020). While stigma is often highlighted as a key explanatory factor, situating these findings within a broader social inclusion framework suggests that it represents one element of a wider system of structural and sociocultural exclusion (Henderson *et al.*, 2026). Consistent income inequalities, limited labour market access and insecure housing conditions indicate that material and institutional factors play a central role in shaping inclusion trajectories.

Integrating evidence from the meta-analysis, thematic synthesis and wider literature highlights the interdependence of social relationships, community participation and socioeconomic conditions in shaping social inclusion among individuals with mental disorders (Boardman, 2011). These processes were further shaped by intersecting axes of inequality, including gender and ethnicity, operating with socioeconomic conditions to produce cumulative and intensifying disparities (Popay, 2010).

More broadly, the literature consistently demonstrated a strong association between socioeconomic inequalities and community isolation; economic disadvantage limits individuals’ ability to participate in social activities and undermines their social roles and sense of self-efficacy. In line with this evidence and the findings of the present review, social exclusion emerged as both driven by – and manifested through – unequal access to essential resources and services (Popay, 2010; Marmot, 2018; Marchi *et al.*, 2024). Recent critical debates have underscored the importance of conceptualising the ‘social’ in mental health as a historically situated and politically constituted construct, to counter reductionist approaches that prioritise individual-level risk factors while insufficiently accounting for broader inequalities and power structures (Bemme and Béhague, 2024).

These findings are consistent with frameworks conceptualising social exclusion as arising from sociocultural dynamics and structural conditions rather than from individual deficits alone (Boardman, 2011). Building on this view, social models of disability provide a coherent interpretative lens by framing exclusion as the product of environmental and relational barriers, rather than individual impairments (Salzer, 2021). In turn, the findings suggest that approaches focusing exclusively on individual-level interventions may be insufficient to address the broader conditions shaping exclusion.

### Integrating objective and subjective dimensions of inclusion

Finally, the findings highlight a recurrent discrepancy between objective indicators of participation and subjective experiences of social inclusion, with activity frequency or participation counts not consistently reflecting perceived inclusion or access to meaningful opportunities (Simplican *et al.*, 2015). Reliance on objective indicators alone may therefore overlook fragile and conditional forms of inclusion, in which participation is achieved through concealment or neutralisation of mental disorders (Davey and Gordon, 2017). Consistent with this, interventions centred primarily on physical presence in the community may reproduce forms of segregation unless accompanied by opportunities for active participation and meaningful social relationships.

These considerations underscore the need for greater integration of qualitative and participatory approaches in social inclusion research in order to better capture lived experience and the perspectives of those directly affected, which may not be fully reflected in objective indicators. This is relevant not only for strengthening conceptual and empirical understanding but also in light of calls for greater epistemic justice in mental health research, recognising the importance of legitimising diverse forms of knowledge (Bemme and Béhague, 2024).

### Implications for practice and policy-making

The findings of this review highlight key implications for practice and policy-making. Conceptualising social inclusion as a multidimensional construct calls for equally integrated responses. However, existing interventions addressing the social and economic circumstances of people with mental disorders remain fragmented and unevenly distributed across domains, and tend to be implemented as discrete strategies rather than approaches capable of addressing the cumulative nature of disadvantage (Barnett *et al.*, 2023).

The structural and cross-national patterns of inequality identified in this review suggest that interventions should move beyond a focus on individual adaptation and instead engage with the social contexts in which people live. Evidence from community-based interventions highlights the importance of contextual factors, including service organisation, local resources and broader social environments, indicating the need for interventions that actively reshape, rather than simply respond to, existing conditions (Killaspy *et al.*, 2022).

The findings also support the relevance of an intersectional perspective, as individuals from marginalised communities may experience compounded forms of disadvantage, while current interventions remain insufficiently tailored to these groups (Baldwin *et al.*, 2025). However, limited reporting and the scarcity of stratified analyses constrain understanding of how intervention effects vary across sociodemographic groups, with some evidence suggesting reduced effectiveness among more disadvantaged populations (Greenburgh *et al.*, 2025).

In conclusion, these findings point to the need for more integrated, context-sensitive and equity-oriented interventions and policies.

### Limitations

The included studies showed heterogeneity in measurement approaches and sample characteristics – varying in size, demographic composition and recruitment methods. Moreover, most evidence derived from Western, high-income countries, potentially limiting generalisability of the findings to other socioeconomic and institutional settings.

Although standardised instruments were used, variability in measurement approaches and operationalisation may only partially capture the complexity and contextual nature of social inclusion, particularly given the documented discrepancies between objective participation and perceived inclusion.

Further limitations include the inability to examine the contribution of specific measurement instruments or national contexts to between-study heterogeneity, as each study used a different scale, and no country contributed more than one study. Clinical heterogeneity was also present, with variation in diagnostic composition and reporting of clinical characteristics, which should be considered when interpreting the findings collectively.

The exploration of heterogeneity was limited to clinical characteristics, reflecting the available data but also highlighting a broader limitation of meta-analytic approaches, which may obscure important social and contextual variation. Future research should examine heterogeneity across a wider range of factors, including gender, ethnicity and intersecting forms of disadvantage.

Additionally, the domains identified reflect those operationalised in existing measures and reported by the authors, and other relevant aspects of social inclusion may be underrepresented or subsumed within broader categories.

Finally, the small number of studies precluded formal assessment of publication bias and limited the power of subgroup analyses and meta-regression.

## Conclusions

This systematic review shows that individuals with mental disorders experience substantially lower levels of social inclusion than the general population. Convergent quantitative and narrative evidence indicates that these disparities are structurally patterned and span multiple domains, especially social relationships, community participation, socioeconomic conditions and housing. The findings also highlight a persistent discrepancy between objective indicators of participation and individuals’ subjective experiences of inclusion. Despite methodological heterogeneity, results were consistent in direction and magnitude, underscoring the relevance of structural and relational determinants of social inclusion in mental health.

## Supporting information

10.1017/S2045796026100730.sm001 Pollice et al. supplementary material Pollice et al. supplementary material

## Data Availability

The data supporting the findings of this study are available from the corresponding author upon reasonable request.

## Supplementary material

The supplementary material for this article can be found at https://doi.org/10.1017/S2045796026100730.

## Figure 1 Long description

The flowchart details the identification and screening process of studies via databases and registers in two rounds. First round: - Records identified from databases (n=1237). - Records removed before screening due to duplicate records (n=203), ineligible by automation tools (n=0) and other reasons (n=0). - Records screened (n=1034). - Records excluded (n=869). - Reports sought for retrieval (n=165). - Reports not retrieved (n=3). - Reports assessed for eligibility (n=162). - Reports excluded due to not using a scale of social inclusion (n=143) and language (n=2). - Identification of 9 measurement scales. Second round: - Records identified from databases (n=139). - Records removed before screening due to duplicate records (n=73). - Records screened (n=66). - Records excluded (n=56). - Reports sought for retrieval (n=10). - Reports not retrieved (n=0). - Reports assessed for eligibility (n=10). - Reports of included studies (n=10). - Reports of included studies for the meta-analysis (n=6). Source: Page MJ, et al. BMJ 2021;372:n71. doi: link.

Navigate back to Figure 1.

## Figure 2 Long description

Author(s) and Year Aubry and Myner, 1996. Individuals with Mental Disorders: n 51, mean 22, sd 8.35. General Population: n 51, mean 29.44, sd 9.88. Weight (percent) 15.4. Standardized Mean Difference [95 percent CI] minus 0.81 [minus 1.21, minus 0.40]. Cabral et al., 2018. Individuals with Mental Disorders: n 183, mean 93.75, sd 19.67. General Population: n 228, mean 104.08, sd 19.67. Weight (percent) 18.3. Standardized Mean Difference [95 percent CI] minus 0.52 [minus 0.72, minus 0.33]. Filia et al., 2022. Individuals with Mental Disorders: n 239, mean 57.48, sd 22.96. General Population: n 267, mean 87.17, sd 11.69. Weight (percent) 18.2. Standardized Mean Difference [95 percent CI] minus 1.65 [minus 1.86, minus 1.45]. Huxley et al., 2012. Individuals with Mental Disorders: n 43, mean 3.95, sd 1.62. General Population: n 212, mean 5.13, sd 1.08. Weight (percent) 16.4. Standardized Mean Difference [95 percent CI] minus 0.93 [minus 1.33, minus 0.65]. Mezey et al., 2020. Individuals with Mental Disorders: n 28, mean 45.5, sd 10.6. General Population: n 28, mean 53.9, sd 9.9. Weight (percent) 13.1. Standardized Mean Difference [95 percent CI] minus 0.81 [minus 1.35, minus 0.26]. Nagata et al., 2020. Individuals with Mental Disorders: n 300, mean 32.63, sd 40.32. General Population: n 300, mean 62.67, sd 50.58. Weight (percent) 18.6. Standardized Mean Difference [95 percent CI] minus 0.68 [minus 0.82, minus 0.49]. A horizontal axis labeled Standardized Mean Difference with tick labels minus 2, 0 and 2. A vertical reference line at 0. Text above the plot area reads Lower SI on the negative side and Higher SI on the positive side. A pooled-effect diamond is shown with Standardized Mean Difference [95 percent CI] minus 0.91 [minus 1.26, minus 0.56]. Heterogeneity: Tau superscript 2 equals 0.1624; Chi superscript 2 equals 76.47, df equals 5, P less than 0.0001; I superscript 2 equals 90.8 percent.

Navigate back to Figure 2.

## Table 1 Long description

The table summarises 10 studies from Canada, Portugal, Australia, the United Kingdom, Hong Kong, Brazil, and the United States that assess community or social inclusion using several named scales. Most studies compare people with mental disorders to general-population samples, and one compares them to siblings. Across measures, findings consistently indicate lower social inclusion for people with mental disorders, often alongside lower social integration and quality of life. Australian studies using the Filia Social Inclusion Measure report lower inclusion across domains, with young adults showing reduced work and education participation, weaker relationships, and more housing instability; young people with psychosis are described as particularly disadvantaged. United Kingdom studies using the Social and Community Opportunities Profile report lower satisfaction with opportunities among service users, and cross-cultural results place the UK patient sample lowest, the Hong Kong patient sample in the middle, and the UK general population highest. The Social Inclusion Questionnaire study reports a marked reduction compared with siblings, especially for social integration and productivity, and the US participation measure study reports lower community participation among those with severe mental disorders. One Portuguese validation study notes lower inclusion across domains except community support, and an international comparison highlights that structural inequalities such as education, income, gender, and ethnicity strongly shape inclusion outcomes. Because the studies use different instruments and aims, results are best interpreted as a consistent directional pattern rather than directly comparable scores across countries or scales.

Navigate back to Table 1.

## Table 2 Long description

The table summarises study samples by reference and country, listing sample sizes and available clinical, general-population, and demographic characteristics for both a mental-disorder group and a comparison group. Sample sizes range from small paired groups of about fifty (Aubry and Myner, 1996) to large matched groups of three hundred (Nagata et al., 2020), with several studies in between. Clinical groups are typically described as severe mental disorders, often clinician diagnosed through services or records, with common diagnoses including schizophrenia, mood disorders, depression, anxiety, bipolar disorder, and personality disorders; some studies also report service history such as hospital stay length, admissions, or years in contact. Where demographics are reported, ages are generally adult and often similar between clinical and comparison groups, though some studies focus on young adults aged eighteen to twenty five or split participants into under and over twenty six. Gender distributions are frequently close between groups, commonly around half to three quarters female, with a few subgroup differences such as carers versus community members in one Australian study. Ethnicity is often not reported, but when included it varies by country, for example mostly White in the United Kingdom samples and more diverse distributions in the United States and Brazil. Several entries note missing information for diagnosis, general-population details, or demographics, so cross-study comparisons should be interpreted cautiously.

Navigate back to Table 2.

## Table 3 Long description

The table summarises research findings across five social inclusion domains for individuals with mental disorders, compared mainly with the general population and sometimes with unaffected siblings or family members. In social connections, studies report weaker and less supportive networks, rare contact with neighbours, greater reliance on digital interaction, and markedly lower perceived friend support among young people with severe conditions; longitudinal evidence suggests the social integration gap can widen over time and may appear before clinical onset. Family support is described as strongly shaped by socioeconomic disadvantage, with lower income and education linked to reduced acceptance and support. For community integration and participation, the largest and most consistent gap is lower engagement and narrower, less diverse participation; adjusting for material conditions reduces some differences but does not remove them, and subjective satisfaction with participation remains poorer even when objective indicators look similar. Structural inequalities related to gender, race or ethnicity, education, and income are reported to compound exclusion and create stratified participation patterns; family members often fall between affected individuals and the general population. In income, employment, and education, studies describe lower earnings, greater financial strain, restricted leisure and social activity due to hardship, poorer employment opportunities often involving sheltered work, worsening productivity over time relative to siblings, and lower formal educational participation among young adults with severe disorders. In housing, individuals are more likely to live alone, report neighbourhood dissatisfaction and greater exposure to crime, and young people with severe disorders show higher likelihood of living with parents and experiencing housing instability; disadvantaged neighbourhood congregate settings may provide shelter without fostering neighbour relationships. Political and civic inclusion is the exception, with one longitudinal study reporting no meaningful differences from unaffected siblings in access to public services or political participation. Findings come from multiple studies and measures, so results should be interpreted as a synthesis of patterns rather than a single standardized metric.

Navigate back to Table 3.
