# Supplementary material for: Social inclusion inequalities between individuals with mental disorders and the general population: a systematic review and meta-analysis
Source: Epidemiol Psychiatr Sci. 2026 Jun 10;35:e35. doi: 10.1017/S2045796026100730 (PMC13276723; doi:10.1017/S2045796026100730)
Supplement: Pollice et al. supplementary material [file S2045796026100730sup001.docx]

## Supplementary Material of the paper “Social Inclusion Inequalities Between Individuals with Mental Disorders and the General Population: A Systematic Review and Meta-Analysis”

### Search query

| Supp. Table 1. Search query | | |  |
| --- | --- | --- | --- |
| DATABASE |  | **QUERY** | **N° reports** |
| PUBMED | 1^st^ | ("social inclusion" OR "social inclusion"[mh] OR "social exclusion"[tiab] OR social isolation [mh] OR "community participation"[mh] OR "community participation" [tiab] OR "public participation" [tiab] OR "community integration" [mh] OR "social integration" [mh] OR "social isolation" [tiab] OR "social marginalization"[mh] OR "social capital"[mh] OR "social participation"[mh] OR "social alienation"[mh] OR "social retreat" [tiab]) AND  (psychometrics[mesh] OR psychometric*[tiab] OR "rating scale"[tiab] OR "Measurement scale"[tiab] OR "assessment tool"[tiab] OR measure* [ti] OR test [tiab] OR scale [ti] OR "validation" [tiab])  AND  (Schizophrenia Spectrum and Other Psychotic Disorders [mh]) | 201 |
|  | 2^nd^ | *“name of the scale”* AND ("mental disorder*"[tiab] OR "psychiatric disorder*"[tiab]  OR "mental illness"[tiab] OR "severe mental illness"[tiab]  OR psychosis[tiab] OR psychotic[tiab] OR schizophrenia[tiab]  OR "bipolar disorder"[tiab] OR "affective disorder*"[tiab]  OR "personality disorder*"[tiab] OR "mood disorder*"[tiab]) | 51 |
| EMBASE | 1^st^ | ('social inclusion'/exp OR 'social exclusion'/exp OR 'social isolation'/exp OR 'community participation'/exp OR 'public participation'/exp OR 'community integration'/exp OR 'social marginalization'/exp OR 'social capital'/exp OR 'social participation'/exp OR 'social alienation'/exp OR 'social retreat') AND ('psychometrics'/exp OR psychometric* OR 'rating scale':ti,ab,kw OR 'measurement scale':ti,ab,kw OR 'assessment tool':ti,ab,kw OR measure*:ti,kw OR 'questionnaire':ti,ab,kw) AND ('schizophrenia spectrum disorder'/exp OR 'psychotic disorder'/exp OR 'psychosis'/exp OR 'schizophrenia'/exp) NOT ('child'/exp OR 'preschool child'/exp OR 'infant'/exp OR 'newborn'/exp) NOT 'aged'/exp | 242 |
|  | 2^nd^ | ('sinque':ti,ab OR 'social inclusion questionnaire':ti,ab OR 'activity and participation questionnaire':ti,ab OR 'community integration measure':ti,ab OR 'community inclusion scale':ti,ab OR 'community inclusion scale for people with psychiatric problems':ti,ab OR 'cis-app':ti,ab OR 'filia social inclusion measure':ti,ab OR 'f-sim':ti,ab OR 'social and community opportunities profile':ti,ab OR 'social inclusion scale':ti,ab OR 'temple university community participation measure':ti,ab OR 'tucp':ti,ab) AND ('mental disorder*':ti,ab OR 'psychiatric disorder*':ti,ab OR 'mental illness':ti,ab OR 'severe mental illness':ti,ab OR psychosis:ti,ab OR psychotic:ti,ab OR schizophrenia:ti,ab OR 'bipolar disorder':ti,ab OR 'affective disorder*':ti,ab OR 'personality disorder*':ti,ab OR 'mood disorder*':ti,ab) | 29 |
| SCOPUS | 1^st^ | ( TITLE-ABS-KEY ( "social inclusion" OR "social exclusion" OR "social isolation" OR "community participation" OR "public participation" OR "community integration" OR "social integration" OR "social marginalization" OR "social capital" OR "social participation" OR "social alienation" OR "social retreat" ) ) AND ( TITLE-ABS-KEY ( psychometric* OR "rating scale" OR "measurement scale" OR "assessment tool" ) OR TITLE ( measure* OR measuring OR test OR scale OR “questionnaire”) ) AND TITLE-ABS-KEY ( "schizophrenia spectrum disorder" OR "psychotic disorder" OR "schizophrenia" OR "schizoaffective disorder" OR "delusional disorder" OR "brief psychotic disorder" OR "schizophreniform disorder" ) AND NOT ( TITLE-ABS-KEY ( "child*" OR "preschool*" OR "infant*" OR "neonatal*" OR "pediatric*" OR "newborn*" ) ) AND NOT ( TITLE-ABS-KEY ( "elderly" OR "older adults" OR "geriatric*" OR "senior*" OR "aged 65+" OR "very old" OR "octogenarian*" OR "nonagenarian*" ) ) | 363 |
|  | 2^nd^ | TITLE-ABS-KEY ( "Sinque" OR "Social Inclusion Questionnaire" OR "Activity and Participation Questionnaire" OR "Community Integration Measure" OR "Community Inclusion Scale" OR "Community Inclusion Scale for People with Psychiatric Problems" OR "Filia Social Inclusion Measure" OR "Social and Community Opportunities Profile" OR "Social Inclusion Scale" OR "Temple University Community Participation Measure" ) AND TITLE-ABS-KEY ( "mental disorder*" OR "psychiatric disorder*" OR "mental illness" OR "severe mental illness" OR psychosis OR psychotic OR schizophrenia OR "bipolar disorder" OR "affective disorder*" OR "personality disorder*" OR "mood disorder*" ) | 36 |
| CINHAL | 1^st^ | ( MH "Social Inclusion" OR MH "Social Exclusion" OR MH "Social Isolation" OR MH "Community Participation" OR MH "Public Participation" OR MH "Community Integration" OR MH "Social Integration" OR MH "Social Marginalization" OR MH "Social Capital" OR MH "Social Participation" OR MH "Social Alienation" OR ("social inclusion" OR "social exclusion" OR "social isolation" OR "community participation" OR "public participation" OR "community integration" OR "social integration" OR "social marginalization" OR "social capital" OR "social participation" OR "social alienation" OR "social retreat") TI OR AB ) AND ( MH "Psychometrics" OR TI ("psychometric*" OR "rating scale" OR "measurement scale" OR "assessment tool" OR "measure*" OR "test" OR "scale" OR "validation" OR "questionnaire") OR AB ("psychometric*" OR "rating scale" OR "measurement scale" OR "assessment tool" OR "measure*" OR "test" OR "scale" OR "validation") ) AND ( MH "Schizophrenia Spectrum Disorders" OR MH "Psychotic Disorders" OR TI ("schizophrenia spectrum disorder" OR "psychosis" OR "psychotic disorder" OR "schizoaffective disorder" OR "delusional disorder") OR AB ("psychosis" OR "schizophrenia spectrum disorder" OR "psychotic disorder" OR "schizoaffective disorder" OR "delusional disorder" OR "brief psychotic disorder") ) | 92 |
|  | 2^nd^ | XB (XB ("Sinque" OR "Social Inclusion Questionnaire"OR "Activity and Participation Questionnaire" OR "APQ6" OR "Community Integration Measure" OR "Community Inclusion Scale" OR "Community Inclusion Scale for People with Psychiatric Problems" OR "Filia Social Inclusion Measure" OR "F-SIM"OR "Social and Community Opportunities Profile"OR "Social Inclusion Scale"OR "Temple University Community Participation Measure" OR "TUCP")) AND XB (("mental disorder*" OR "psychiatric disorder*" OR "mental illness" OR "severe mental illness"OR psychosis OR psychotic OR schizophrenia OR "bipolar disorder" OR "affective disorder*"OR "personality disorder*" OR "mood disorder*")) | 13 |
| PSYCINFO | 1^st^ | ("social inclusion" OR "social exclusion" OR "social integration" OR "community engagement" OR "social capital" "social marginalization" OR "social participation" OR "social alienation" OR "social retreat") AND (psychometric* OR (TI( assessment tools or assessment method or assessing OR evaluation or screening or test or measuring or measure*)) OR (AB ( assessment tools or assessment method or assessing OR evaluation or screening or measuring or measure*)))AND (psychosis or "psychotic disorder" or psychotic or schizophrenia) | 339 |
|  | 2^nd^ | XB (XB ("Sinque" OR "Social Inclusion Questionnaire"OR "Activity and Participation Questionnaire" OR "APQ6" OR "Community Integration Measure" OR "Community Inclusion Scale" OR "Community Inclusion Scale for People with Psychiatric Problems" OR "Filia Social Inclusion Measure" OR "F-SIM"OR "Social and Community Opportunities Profile"OR "Social Inclusion Scale"OR "Temple University Community Participation Measure" OR "TUCP")) AND XB (("mental disorder*" OR "psychiatric disorder*" OR "mental illness" OR "severe mental illness"OR psychosis OR psychotic OR schizophrenia OR "bipolar disorder" OR "affective disorder*"OR "personality disorder*" OR "mood disorder*")) | 18 |

1. Selection and assessment of social inclusion measures

The identification of social inclusion measures was guided by a conceptual and multidisciplinary approach. Instruments were assessed by a multidisciplinary team, including mental health researchers, clinicians, social scientists, and a researcher with lived experience, with disagreements resolved through discussion and consensus. Instruments were included if they conceptualised social inclusion as a multidimensional construct (Cordier *et al.* 2017), while measures focusing exclusively on single indicators (e.g. housing, education, or social connections alone) were excluded, as these were considered partial proxies rather than multidimensional measures of social inclusion. Eligible instruments were required to capture more than one domain within a broader conceptual framework. Measures of specific domains were included when based on validated instruments conceptualised within the social inclusion framework. Identified measures were cross-checked against existing systematic reviews of social inclusion instruments to enhance consistency (Coombs *et al.* 2013; Cordier *et al.* 2017). Only instruments with evidence of psychometric validation in the target population were included. Validation studies were identified through the initial search and a parallel review of social inclusion measures.

### Reason for exclusion from meta-analysis

| SUPP. TABLE 2. REASON FOR EXCLUSION FROM META-ANALYSIS | |
| --- | --- |
| *Study* | **Reason for exclusion from meta-analysis** |
| *Huxley et al. 2016* | UK samples already used for another included study (Huxley *et al.* 2012) |
| *Santos et al. 2018* | UK samples already used for another included study (Huxley *et al.* 2012) |
| *Filia et al. 2022* | No available data on mean and standard deviations |
| *Gardner et al. 2019* | No available data on mean and standard deviations |

### Retrieved measures of social inclusion

| SUPP. TABLE 3. RETRIEVED MEASURES OF SOCIAL INCLUSION | | |  |  |
| --- | --- | --- | --- | --- |
| Name of the scale | **Author, year** | **Country** | **Psychometric validation** | **Domains** |
| Activity and Participation Questionnaire (APQ6) | Stewart *et al.* 2010 | Australia | (Stewart *et al.* 2010) | Employment, education, non-paid work, community activities |
| Community Integration Measure (CIM) | McColl, 2001 | Canada | (Shioda *et al.* 2017) | Community participation and belonging, acceptance, social connections, self-determination |
| Community Inclusion Scale (CIS) | Aubry & Myner, 1996 | Canada | (Aubry & Myner, 1996) | Community integration and social contacts |
| Community Inclusion Scale for People with Psychiatric Problems (CIS-APP) | Cabral *et al.* 2014 | Portugal | (Cabral *et al.* 2018) | Community inclusion and belonging, social activities, social contacts and independence |
| Filia Social Inclusion Measure (F-SIM) | Filia *et al.* 2019 | Australia | (K. Filia *et al.* 2022; K. M. Filia *et al.* 2019) | Housing & Neighbourhood, social connections, employment & education, finances, wellbeing |
| Social and Community Opportunities Profile (SCOPE) | Huxley *et al.* 2012 | UK | (Huxley *et al.* 2012) | Housing & Neighbourhood, community activities, social connections, employment & education, finances, political participation |
| Social Inclusion Questionnaire (SINQUE) | Mezey *et al.* 2014 | UK | (Mezey *et al.* 2020) | Housing & Neighbourhood, social connections, employment & education, finances, political participation |
| Social Inclusion Scale (SIS) | Secker *et al.* 2009 | USA | (Wilson & Secker, 2015) | Community inclusion, social belonging and acceptance, social connections |
| Temple University Community Participation Measure (TUCP) | Salzer *et al.* 2014 | USA | (Salzer *et al.* 2014, 2015) | Community inclusion, political participation, employment, education |

### 5. Risk of bias and GRADE

Overall, studies exhibited low to moderate risk of bias, mainly due to sampling differences, with outcome measurement judged consistently low risk.

As all included studies were observational, the certainty of evidence started at a low level. It was downgraded for risk of bias, due to non-random sampling and limited comparability between individuals with mental disorders and the general population, and for inconsistency, reflecting substantial between-study heterogeneity (I² = 90.8%). No downgrades were applied for indirectness or imprecision, as all studies used validated measures and confidence intervals did not include the null value. Publication bias could not be formally assessed because of the small number of studies included. The certainty of the evidence was upgraded owing to the large and directionally consistent pooled effect. Overall, the certainty of the evidence was judged to be low.

| SUPP. TABLE 4. GRADE | |  |
| --- | --- | --- |
| Domain | **Judgement** | **Explanation** |
| *Risk of bias* | Serious (–1) | Most studies used non-random sampling, with individual with mental disorder recruited through clinical services and general population groups through community convenience samples. Although outcome measurement was consistent across groups, selection processes limit comparability. |
| *Inconsistency* | Serious (–1) | Very high heterogeneity (I² = 90.8%) |
| *Indirectness* | Not serious | Social inclusion was measured using validated multidimensional scales. |
| *Imprecision* | Not serious | The pooled effect size was precise (95% CI: −1.26 to −0.56) and did not include the null value. |
| *Publication bias* | Not applicable |  |
| *Other considerations* | Large effect (+1) | The pooled SMD (−0.91) represents a large and consistent effect across studies, robust to sensitivity analyses. |

### 6. Leave-one-out analyses

Leave-one-out analyses confirmed the robustness of the pooled estimate, with SMDs ranging from –0.70 to –1.00 (all estimates with 95% CIs excluding the null) across iterations and minimal fluctuation in the overall effect size. Heterogeneity estimates remained stable (I² ≈ 89%–93%), except for Filia *et al.* (2022), whose exclusion substantially reduced heterogeneity (I² = 43%) while preserving a significant effect (SMD = –0.70, 95% CI: –0.87; –0.54). These findings indicate that, although this study contributed disproportionately to heterogeneity, the pooled effect was not driven by any single study.

**References**

Aubry, T., & Myner, J. (1996). Community Integration and Quality of Life: A Comparison of Persons With Psychiatric Disabilities in Housing Programs and Community Residents Who Are Neighbours. *Canadian Journal of Community Mental Health*, *15*(1), 5–20. https://doi.org/10.7870/cjcmh-1996-0001

Cabral, J., Carvalho, C. B., da Motta, C., & Sousa, M. (2018). Validation of the Community Integration Scale for Adults with Psychiatric Disorders (CIS-APP-34). *Community Mental Health Journal*, *54*(5), 673–681. https://doi.org/10.1007/s10597-017-0228-2

Coombs, T., Nicholas, A., & Pirkis, J. (2013). A review of social inclusion measures. *The Australian and New Zealand Journal of Psychiatry*, *47*(10), 906–919. https://doi.org/10.1177/0004867413491161

Cordier, R., Milbourn, B., Martin, R., Buchanan, A., Chung, D., & Speyer, R. (2017). A systematic review evaluating the psychometric properties of measures of social inclusion. *PLOS ONE*, *12*(6), e0179109. https://doi.org/10.1371/journal.pone.0179109

Filia, K., Gao, C. X., Jackson, H. J., Menssink, J., Watson, A., Gardner, A., Cotton, S. M., & Killackey, E. (2022). Psychometric properties of a brief, self-report measure of social inclusion: The F-SIM16. *Epidemiology and Psychiatric Sciences*, *31*, e8. https://doi.org/10.1017/S2045796021000755

Filia, K. M., Jackson, H. J., Cotton, S. M., & Killackey, E. J. (2019). Developing and testing the F-SIM, a measure of social inclusion for people with mental illness. *Psychiatry Research*, *279*, 1–8. https://doi.org/10.1016/j.psychres.2019.06.038

Huxley, P., Evans, S., Madge, S., Webber, M., Burchardt, T., McDaid, D., & Knapp, M. (2012). Development of a social inclusion index to capture subjective and objective life domains (Phase II): Psychometric development study. *Health Technology Assessment*, *16*(01), 1–248. https://doi.org/10.3310/hta16010

Mezey, G., White, S., Harrison, I., Bousfield, J., Lloyd-Evans, B., Payne, S., & Killaspy, H. (2020). Validity, reliability, acceptability, and utility of the Social Inclusion Questionnaire User Experience (SInQUE): A clinical tool to facilitate social inclusion amongst people with severe mental health problems. *Social Psychiatry & Psychiatric Epidemiology*, *55*(7), 953–964. CINAHL Plus with Full Text. https://doi.org/10.1007/s00127-019-01826-3

Salzer, M. S., Brusilovskiy, E., Prvu-Bettger, J., & Kottsieper, P. (2014). Measuring community participation of adults with psychiatric disabilities: Reliability of two modes of data collection. *Rehabilitation Psychology*, *59*(2), 211–219. Scopus. https://doi.org/10.1037/a0036002

Salzer, M. S., Kottsieper, P., & Brusilovskiy, E. (2015). Intermethod reliability and factors affecting recall with the Temple University Community Participation measure. *Journal of Mental Health*, *24*(4), 189–195. Scopus. https://doi.org/10.3109/09638237.2015.1036976

Shioda, A., Tadaka, E., & Okochi, A. (2017). Reliability and validity of the Japanese version of the Community Integration Measure for community-dwelling people with schizophrenia. *International Journal of Mental Health Systems*, *11*(1), 29. https://doi.org/10.1186/s13033-017-0138-2

Stewart, G., Sara, G., Harris, M., Waghorn, G., Hall, A., Sivarajasingam, S., Gladman, B., & Mowry, B. (2010). A brief measure of vocational activity and community participation: Development and reliability of the activity and participation questionnaire. *Australian and New Zealand Journal of Psychiatry*, *44*(3), 258–266. Scopus. https://doi.org/10.3109/00048670903487175

Wilson, C., & Secker, J. (2015). *Validation of the Social Inclusion Scale with Students | Article | Social Inclusion*. https://www.cogitatiopress.com/socialinclusion/article/view/121
